# Impaired Insulin/IGF Signaling in Experimental Alcohol-Related Myopathy 

**DOI:** 10.3390/nu4081058

**Published:** 2012-08-20

**Authors:** Van Anh Nguyen, Tran Le, Ming Tong, Elizabeth Silbermann, Fusun Gundogan, Suzanne M. de la Monte

**Affiliations:** 1 Department of Medicine, Rhode Island Hospital, Alpert Medical School of Brown University, 55 Claverick Street, Providence, RI 02903, USA; Email: va.nguyen87@gmail.com (V.A.N.); tran.le09@gmail.com (T.L.); ming_tong_min@yahoo.com (M.T.); 2 Department of Neurology, Rhode Island Hospital, Alpert Medical School of Brown University, 593 Eddy Street, Providence, RI 02903, USA; Email: Elizabeth_Silbermann@Brown.edu; 3 Department of Pathology, Division of Perinatal Pathology, Women and Infants Hospital of Rhode Island, Alpert Medical School of Brown University, 55 Claverick Street, Providence, RI 02903, USA; Email: Fusun_Gundogan@Brown.edu; 4 Departments of Neuropathology/Pathology, Neurology, Neurosurgery, and Medicine, Rhode Island Hospital, Alpert Medical School of Brown University, 55 Claverick Street, Providence, RI 02903, USA

**Keywords:** alcohol, myopathy, insulin resistance, signal transduction, experimental model, multiplex ELISA, Akt pathway, gene expression, acetylcholine, oxidative stress, mitochondrial dysfunction

## Abstract

Alcohol-related myopathy (Alc-M) is highly prevalent among heavy drinkers, although its pathogenesis is not well understood. We hypothesize that Alc-M is mediated by combined effects of insulin/IGF resistance and oxidative stress, similar to the effects of ethanol on liver and brain. We tested this hypothesis using an established model in which adult rats were pair-fed for 8 weeks with isocaloric diets containing 0% (*N* = 8) or 35.5% (*N* = 13) ethanol by caloric content. Gastrocnemius muscles were examined by histology, morphometrics, qRT-PCR analysis, and ELISAs. Chronic ethanol feeding reduced myofiber size and mRNA expression of IGF-1 polypeptide, insulin, IGF-1, and IGF-2 receptors, IRS-1, and IRS-2. Multiplex ELISAs demonstrated ethanol-associated inhibition of insulin, IRS-1, Akt, and p70S6K signaling, and increased activation of GSK-3β. In addition, ethanol-exposed muscles had increased 4-hydroxy-2-nonenal immunoreactivity, reflecting lipid peroxidation, and reduced levels of mitochondrial Complex IV, Complex V, and acetylcholinesterase. These results demonstrate that experimental Alc-M is associated with inhibition of insulin/IGF/IRS and downstream signaling that mediates metabolism and cell survival, similar to findings in alcoholic liver and brain degeneration. Moreover, the increased oxidative stress, which could be mediated by mitochondrial dysfunction, may have led to inhibition of acetylcholinesterase, which itself is sufficient to cause myofiber atrophy and degeneration.

## 1. Introduction

### 1.1. Overview

Alcohol abuse is a leading cause of morbidity and mortality world-wide [1]. In the United States alone, alcohol abuse adds billions to annual healthcare costs due to disabilities resulting from neuropsychiatric disorders, stroke, dementia, cardiovascular disease, peripheral neuropathy, and liver disease [2]. The true magnitude of this problem is further exposed by the co-factor role heavy alcohol abuse plays in the pathogenesis of colorectal, lung, breast, head and neck cancers, neurodevelopmental disorders, accidents, family fall-outs, and socioeconomic failures [3]. 

### 1.2. Acute and Chronic Alcohol-Related Myopathy

Myopathy is an under-appreciated consequence of chronic alcohol abuse. Acute alcoholic myopathy, which occurs in up to 5% of chronic heavy drinkers, is characterized by rhabdomyolysis [4] with extensive myofiber necrosis, phagocytosis, loss of A-band structure (thick filament zone), and myofiber regeneration [5,6,7]. Eventually, myofibers shrink and central nuclei increase in abundance. The pathogenesis of acute alcoholic myopathy is unknown, although factors such as ischemia, potassium or phosphate depletion, and direct toxic effects of alcohol or acetaldehyde have been considered.

Chronic myopathic myopathy, characterized by progressive weakness in proximal muscles [8,9,10], occurs in 33% to 67% of alcoholics [6,7,10]. Afflicted individuals develop fatigue with difficulty climbing stairs, walking, and rising from squatted and seated positions [6]. The patterns of weakness can be alcohol dose-dependent, and abate or resolve with abstinence [11]. A diagnosis of primary myopathy in alcoholics can be confirmed by electromyography [12]. In addition, the main histopathological features include, selective atrophy of glycolytic, fast-twitch (Type 2) myofibers, compensatory hypertrophy of Type 1 (aerobic) myofibers [6,13], and scattered myofibers with moth-eaten appearances in sections stained for oxidative enzyme activity. Myofiber necrosis, inflammation, membrane damage, and fibrosis are generally absent. Progressive myofiber atrophy leads to wasting with up to 30% loss of muscle mass [11]. Ultrastructural studies revealed increased lipid deposition, dilatation of the sarcoplasmic reticulum, loss of myofilaments [6,14], and distortion of mitochondrial cristae in human alcohol-related myopathy (Alc-M) [15]. The striking similarities in the ultrastructural and biochemical pathology in chronic Alc-M and chronic alcoholic steatohepatitis [16,17] suggest that these disease processes may share pathogenic mechanisms. 

### 1.3. Oxidative Stress as a Potential Mediator of Alc-M

Relatively little is known about the degenerative processes that lead to myopathic myopathy in alcoholics. One potential mediator is oxidative stress caused by ethanol or acetaldehyde. Build-up of reactive oxygen species (ROS) and free radicals can lead to adduct formation with proteins, RNA, DNA, and lipids, thereby impairing critical cellular functions [14,15,18]. In addition, acetaldehyde compromises intra-cellular anti-oxidant mechanisms by inhibiting superoxide dismutase and glutathione peroxidase, resulting in increased levels of malondialdehyde (MDA) [15]. Lipid peroxidation-mediated tissue damage destroys the structural and functional integrity of cell membranes and causes mitochondrial dysfunction [14]. The greater vulnerability of Type 2 myofibers in Alc-M could be due to their inherently lower levels of anti-oxidant mechanisms compared with Type 1 myofibers [15]. This concept is supported in part by the finding that selenium and α-tocopherol levels are reduced in skeletal muscles of chronic alcohol-exposed experimental animals [19]. On the other hand, the failure of α-tocopherol (Vitamin E), zinc or selenium supplements to reverse Alc-M [20] suggests that oxidative stress is not the sole mediator of this disease. 

### 1.4. Malnutrition as a Contributing Factor in Alc-M

Malnutrition is nearly always suggested as a potential cause of Alc-M because 40% of alcoholics have nutritional deficiencies [15]. However, there is little convincing evidence that favors a role for micro-nutrient deficiencies in Alc-M pathogenesis. Instead, blood levels of Vitamins D, B1 (thiamine), B2 (riboflavin), B6 (pyridoxine), B9 (folate) and B12 (cobalamins) were found to be similar in alcoholics with or without myopathy [21]. Moreover, Vitamin B9 and B12 supplementation trials did not significantly alter clinical symptoms of Alc-M [15]. On the other hand, a role for macronutrient deficiency as a cause for Alc-M has not been sufficiently evaluated.

### 1.5. Alc-M Maybe Mediated by Insulin and Insulin-Like Growth Factor Resistance

Since ultrastructural and biochemical abnormalities in chronic Alc-M and alcoholic steatohepatitis are shared, we hypothesize that insulin and insulin-like growth factor (IGF) resistance, which mediates chronic alcoholic liver disease [22,23,24], also contributes to the pathogenesis of chronic Alc-M. This concept is supported by the findings that: (1) insulin regulates gene and protein expression, energy metabolism, glucose uptake, and glucose utilization [25]; (2) ethanol impairs glucose uptake and utilization, insulin-stimulated glycogen phosphorylase kinase activity [26], and insulin sensitivity; (3) ethanol reduces circulating levels of IGF-1 [25,27,28], which is a major trophic factor for skeletal muscle [29]; and (4) ethanol toxicity promotes mitochondrial dysfunction, oxidative stress, and pro-apoptosis signaling [30], all of which are features of alcoholic myopathy. Moreover, *in vitro* experiments confirmed that ethanol inhibits glucose uptake and utilization in skeletal muscle [31]. This suggests that the increased glycogen deposits in alcoholic skeletal muscle are due to impaired glucose utilization. 

### 1.6. Goals of the Research

In this study, we utilized a robust experimental model of chronic ethanol feeding in which the diets were nutritionally balanced and replete with ample micro- and macronutrients. We assessed the degree to which alcohol-induced myopathic myopathy was mediated by: (1) impaired expression of genes that regulate insulin/IGF signaling; (2) reduced activation of insulin/IGF signaling networks; and (3) increased oxidative stress with mitochondrial dysfunction and adduct formation.

## 2. Experimental Section

### 2.1. Materials

Reagents for preparing isocaloric liquid diets (F1259 and F1258) were purchased from BioServ (Frenchtown, NJ, USA). See detailed formulations in Supplementary Table S1. The bicinchoninic acid (BCA) kit to measure protein concentration was purchased from Pierce Chemical Co. (Rockford, IL, USA). Histochoice fixative was purchased from Amresco, Inc. (Solon, OH, USA). Amplex UltraRed soluble fluorophore and the Akt Pathway Total and Phospho 7-Plex panels were purchased from Invitrogen (Carlsbad, CA, USA). Maxisorp 96-well enzyme-linked immunosorbant assay (ELISA) plates were from Nunc (Thermo Fisher Scientific; Rochester, NY, USA). Horseradish peroxidase (HRP) conjugated antibodies were from Pierce Chemical Co. (Rockford, IL, USA). All other monoclonal antibodies and immunodetection reagents were purchased from Abcam (Cambridge, MA, USA), Proteintech Group, Inc. (Chicago, IL, USA), Invitrogen (Carlsbad, CA, USA) or Percipio Biosciences, Inc. (Burlingame, CA, USA). Fine chemicals were purchased from CalBiochem (Carlsbad, CA, USA), or Sigma-Aldrich (St Louis, MO, USA). QIAzol Lysis Reagent for RNA extraction and QuantiTect SYBR Green PCR Mix were obtained from Qiagen, Inc. (Valencia, CA, USA). The AMV 1st Strand cDNA Synthesis Kit was purchased from Roche Applied Science (Indianapolis, IN, USA). Synthetic oligonucleotides used in quantitative polymerase chain reaction (qPCR) assays were purchased from Sigma-Aldrich Co. (St. Louis, MO, USA). The Stereologer system used for image analysis was purchased from the Stereology Resource Center (Chester, MD, USA).

### 2.2. Chronic Ethanol Exposure Model

Adult male (~200–250 g) Long Evans rats (Harlan Sprague Dawley, Inc., Indianapolis, Indiana) were pair-fed with isocaloric liquid diets containing 0% (*N* = 8) or 35.5% (*N* = 13) caloric content (9.2% v/v) pharmaceutical-grade ethanol for 8 weeks [23]. The diets were nutritionally complete and identical except for the replacement of some carbohydrates with ethanol (Table S1). The rats were adapted to the liquid diets over the 2 weeks prior to starting the experiment. Rats were monitored daily to ensure adequate nutritional intake and maintenance of body weight. Blood alcohol levels were measured at 8 AM using the Analox GM7 apparatus (Analox Instruments USA, Lunenburg, MA, USA). At the end of the experiment, the rats were sacrificed by isofluorane inhalation. Immediately after excision, the gastrocnemius muscles were divided to snap-freeze portions in a dry ice/methanol bath for protein and RNA studies, or fix in Histochoice for histological studies. Fixed samples were embedded in paraffin, and 2 µm thick sections were stained with Hematoxylin and Eosin for morphometric analysis of fiber diameters using the nucleator probe of the Stereologer program (200× magnification). Throughout the experiment, rats were housed under humane conditions and kept on a 12-h light/dark cycle with free access to food. All experiments were performed in accordance with protocols approved by Institutional Animal Care and Use Committee at the Lifespan-Rhode Island Hospital, and they conform to guidelines established by the National Institutes of Health. 

### 2.3. Quantitative Reverse Transcriptase Polymerase Chain Reaction (qRT-PCR) Assays of Gene Expression

Total RNA was isolated from skeletal muscle using the EZ1 RNA Universal Tissue Kit and the BIO Robot EZ1 (Qiagen Inc., Valencia, CA, USA). RNA was reverse transcribed with random oligonucleotide primers and the AMV First Strand cDNA synthesis kit. The resulting cDNAs were used to measure gene expression by qPCR analysis with gene-specific primers [30]. Primers were designed using MacVector 10 software (MacVector, Inc., Cary, NC, USA) and target specificity was verified using NCBI-BLAST (Basic Local Alignment Search Tool). The Master ep-Realplex instrument and software (Eppendorf AG, Hamburg, Germany) were used to detect amplified signals from triplicate reactions. Using the average C_T_ values, the ng levels of mRNA or 18S rRNA were calculated from standard curves generated with known fixed amounts of subcloned target sequences corresponding to the transcripts. Relative mRNA abundance was calculated from the ng ratios of mRNA to 18S rRNA measured in the same samples, and those data were used for inter-group comparisons. Parallel control studies included reactions with: (1) no template; (2) RNA that was not reverse transcribed; (3) RNA samples pre-treated with DNAse I; (4) RNAse A pre-treated RNA (prior to the reverse transcriptase reaction); and (5) genomic DNA. Although mRNA levels can be compared using the 2^−ΔΔCT^ method [32], we elected to calculate relative transcript abundance because 18S rRNA levels very accurately reflect template input [33]. 

### 2.4. Duplex ELISA

Tissues homogenized in radioimmunoprecipitation assay buffer containing protease and phosphatase inhibitors were used in direct binding ELISAs [30]. Insoluble debris was pelleted by centrifuging the samples at 14000× *g* for 10 min. Supernatant proteins were diluted in Tris buffered saline (TBS). Proteins (40 ng/100 µL) were adsorbed to the bottoms of 96-well ELISA plates by over-night incubation at 4 °C. Non-specific sites were blocked by a 3-h room temperature with 3% BSA in Tris buffered saline (TBS). Samples were incubated with primary antibody (0.2–1.0 µg/mL) for 1 h at 37 °C. The antibody sources and target specificities are provided in Table S2. Immunoreactivity was detected with HRP-conjugated secondary antibody (1:10,000) and the Amplex Red soluble fluorophore [30]. Fluorescence was measured (Ex 530/Em 590) in a SpectraMax M5 microplate reader. Subsequently, the samples were incubated with biotin-conjugated antibodies to large ribosomal protein (RPLPO), and immunoreactivity was detected with streptavidin-conjugated alkaline phosphatase (1:1000) and the 4-Methylumbelliferyl phosphate (4-MUP) fluorophore. Fluorescence (Ex 360/Em 450) intensity was measured in a SpectraMax M5 reader. Binding specificity was determined from parallel negative control reactions in which the primary or secondary antibody was omitted. The ratio of specific protein/RPLPO immunoreactivity was calculated and used for inter-group statistical comparisons. Control studies demonstrated no detection of Amplex signals in the Ex/Em settings for 4-MUP, and linear increases in RPLPO/4-MUP with increasing amounts of protein between 10 and 200 ng/well. Moreover, we demonstrated that there was no significant loss of RPLPO/4-MUP signal in duplex ELISAs compared with single-plex ELISAs, *i.e*., RPLPO-4-MUP only was assayed (data not shown). The latter indicates that proteins remained stably bound to the ELISA plates throughout the procedure.

### 2.5. Multiplex ELISA

To assess the integrity of insulin/IGF-1 signaling, we measured immunoreactivity to the insulin receptor (IR), IGF-1 receptor (IGF-1R), IRS-1, Akt, glycogen synthase kinase 3β (GSK-3β), p70S6 Kinase (p70S6K), and PRAS40 using the Total Akt 7-Plex Panel, and pYpY1162/1163-IR, pYpY1135/1136-IGF-1R, pS312-IRS-1, pS473-Akt, pS9-GSK3β, pTpS421⁄424-p70S6K, and pT246-PRAS40 using the Phospho-Akt 7-Plex ELISA Panel according to the manufacturer’s protocol. Fresh frozen skeletal muscle was homogenized in lysis buffer (50 mM Tris-HCl, pH 7.5, 1% Triton X-100, 2 mM EGTA, 10 mM EDTA, 100 mM NaF, 1 mM Na_4_P_2_O_7_, 2 mM Na_3_VO_4_) containing protease and phosphatase inhibitors [30]. Insoluble debris was pelleted by centrifugation at 14,000× *g* for 10 min. Supernatant protein samples (200 µg each) were incubated with the antibody-coated beads. Captured antigens were detected with biotinylated secondary antibodies and phycoerythrin-conjugated Streptavidin. Results were analyzed using a Bio-Plex 200 system (Bio-Rad, Hercules, CA, USA). Data are expressed as fluorescence light units (FLU) corrected for protein concentration. Standard curves were included in all assays to verify the linear dynamic range for immunoreactivity. 

### 2.6. Statistical Analysis

Data depicted in box plots reflect group medians, 95% confidence interval limits and range (whiskers), and tabulated data reflect means ± SEMs for each group. Intergroup comparisons were made using Student *t*-tests. Data were analyzed using GraphPad Prism 5 software (GraphPad Software, Inc., San Diego, CA, USA). Significant *P*-values (<0.05) are shown within the graph panels or tables.

## 3. Results

### 3.1. General Effects of Ethanol Feeding

The control and ethanol-fed rats gained weight continuously throughout the study, and the final mean body weights were similar in the control (423 ± 39.6 g) and ethanol-fed (454.1 ± 18.4 g) groups. As expected, the mean blood alcohol concentration was elevated in ethanol-fed rats (28.2 ± 2.6 mmol/L) and virtually undetectable in controls (0.57 ± 0.19 mmol/L) (*P* < 0.0001). The chronic ethanol feeding caused hyperglycemia (mmol/L) (Control = 5.7 ± 0.32, Ethanol = 9.58 ± 1.62; *P* = 0.032) and hyper-triglyceridemia (mmol/L) (Control = 0.44 ± 0.02, Ethanol = 0.59 ± 0.07; *P* = 0.0021), reflecting effects of insulin resistance. Throughout the experiment, the rats in both groups were in good health, they self-groomed, remained physically active, and exhibited no signs of motor weakness or discomfort. Complete autopsies demonstrated that the chronic ethanol feeding caused steatohepatitis and neurodegeneration, as previously described [22,23], but no evidence of pancreatitis, cardiovascular disease, or gastrointestinal mucosal pathology. 

### 3.2. Experimental Alcohol-Related Myopathy

The muscles were not weighed due to the rapid freezing and fixing protocol implemented to minimize RNA and protein degradation. Nonetheless, macroscopic examination revealed no evidence of muscle wasting in the ethanol-fed group. Histological studies of gastrocnemius muscles revealed relatively uniform myofiber populations in control samples, but increased variation in myofiber size due to myofiber atrophy or hypertrophy in the ethanol-exposed samples (Figure S1). Although denervation myopathy, characterized by individual and small groups of angulated atrophic fibers, was observed in ethanol-fed rats, most of the atrophic myofibers were polygonal shaped and not grouped or clustered. In addition, increased central nuclei and myofiber splitting were observed in alcohol-exposed relative to control muscle. There was no inflammation or myofiber necrosis. Image analysis confirmed that chronic ethanol exposure caused myofiber atrophy as demonstrated by the left-ward skewing of myofiber diameters, and the significantly smaller mean myofiber diameter in ethanol-fed (25.34 ± 4.38 μm) relative to control (44.97 ± 5.31 μm) rats (*P* = 0.0001). Together, these findings support the concept that chronic high-level ethanol-exposures can cause degenerative myopathic myopathy. 

### 3.3. Ethanol Effects on Skeletal Muscle Expression of Insulin/IGF Pathway Genes

**Figure 1:**
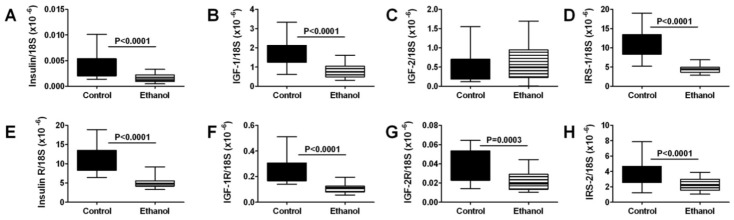
Chronic ethanol feeding impairs insulin/IGF signaling in skeletal muscle. Gastrocnemius muscles from control and chronic ethanol fed rats were used to measure mRNA expression of (**A**) insulin; (**B**) IGF-1; (**C**) IGF-2; (**D**) IRS-1; (**E**) insulin receptor; (**F**) IGF-1 receptor; (**G**) IGF-2 receptor; and (**H**) IRS-2 by qRT-PCR analysis. Gene expression was normalized to 18S rRNA. Inter-group comparisons were made using Student *t*-tests.

We used qRT-PCR analysis to measure the expression of genes that regulate insulin and IGF signaling networks. All samples of gastrocnemius muscle from control and ethanol-fed rats had detectable mRNA levels of insulin, IGF-1, and IGF-2 polypeptides and receptors, IRS-1 and IRS-2 (Figure 1). Among polypeptide genes, IGF-1 was the most abundantly expressed, followed by IGF-2, while insulin was expressed at exceedingly low levels. In contrast, with regard to receptors, insulin receptor expression was most abundant, followed by IGF-1 receptor, and then IGF-2 receptor. IRS-1 was more abundantly expressed than IRS-2. IRS-4 mRNA transcripts were not detected in skeletal muscle. Chronic ethanol feeding significantly reduced the mean mRNA levels of insulin and IGF-1 polypeptides, insulin, IGF-1, and IGF-2 receptors, IRS-1, and IRS-2 (Figure 1). Therefore, within this series, the only mRNA transcript that was not adversely affected by chronic ethanol feeding was IGF-2 polypeptide.

### 3.4. Ethanol Effects on Insulin/IGF Signaling Molecules-Multiplex ELISA Studies

**Figure 2:**
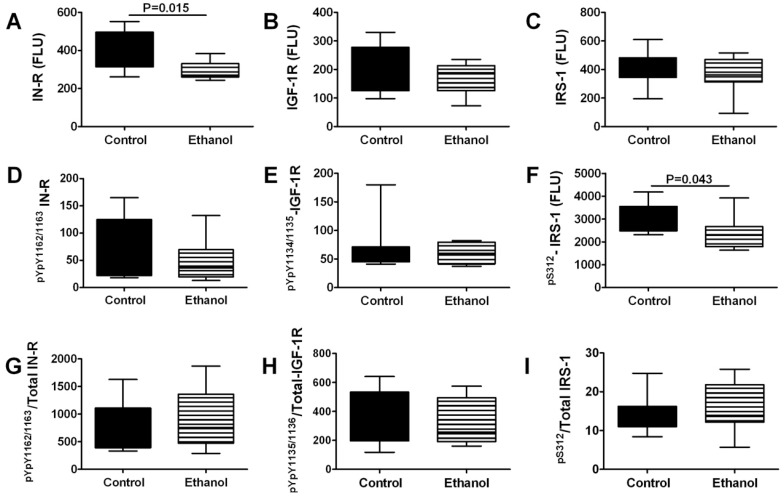
Effects of chronic ethanol feeding on upstream mediators of insulin/IGF signaling networks in skeletal muscle. Gastrocnemius muscles from control and chronic ethanol fed rats were used to measure immunoreactivity to the (**A**) insulin receptor (IN-R); (**B**) IGF-1R; (**C**) IRS-1; (**D**) pYpY1162/1163-IN-R; (**E**) pYpY1135/1136-IGF-1R; (**F**) pS312-IRS-1, and relative levels of phosphorylated (**G**) insulin receptor; (**H**) IGF-1 receptor; and (**I**) IRS-1 using multiplex bead-based ELISA platforms. Immunoreactivity is expressed in fluorescent light units corrected for protein input. Inter-group comparisons were made using Student *t*-tests.

To further characterize the effects of chronic ethanol exposure on insulin and IGF signaling networks we measured insulin receptor, IGF-1 receptor, and IRS-1, and their phosphorylated forms, *i.e*., pYpY1162/1163-IR, pYpY1135/1136-IGF-1R, pS312-IRS-1 in gastrocnemius muscle tissue by Multiplex ELISA. In addition, we calculated the phosphorylated/total protein ratios to assess relative degrees of phosphorylation (Figure 2). Corresponding with the qRT-PCR analyses, chronic ethanol exposure significantly reduced the mean levels of insulin receptor protein expression in gastrocnemius muscle. In contrast, expression levels of IGF-1 receptor and IRS-1 proteins were not significantly altered by the chronic ethanol feeding. Multiplex ELISA studies did not detect significant inter-group differences in the constitutive levels of insulin or IGF-1 receptor phosphorylation, and correspondingly, the relative levels of phosphorylated/total protein were also not significantly altered by ethanol. In contrast, chronic ethanol feeding significantly reduced the mean level of pS312-IRS-1. Although since serine phosphorylation inhibits signaling through IRS-1 [34], recent evidence suggests that pS312-IRS-1 has positive stimulatory effects [35]. Therefore, the significantly reduced levels of pS312-IRS-1 in ethanol-exposed skeletal muscle corroborate the other evidence for global inhibition of insulin/IGF/IRS pathway activation.

### 3.5. Ethanol Impairs Signaling Downstream of Insulin/IGF-IRS-1

**Figure 3:**
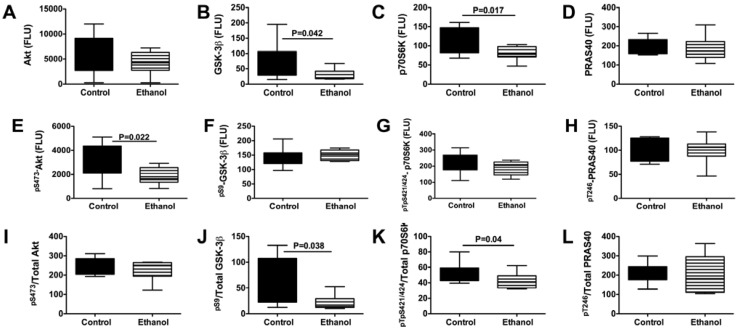
Effects of chronic ethanol feeding on downstream mediators of insulin/IGF signaling networks in skeletal muscle. Gastrocnemius muscles from control and chronic ethanol fed rats were used to measure immunoreactivity to (**A**) Akt; (**B**) glycogen synthase kinase 3β (GSK-3β); (**C**) p70S6 Kinase (p70S6K); (**D**) PRAS40; (**E**) pS473-Akt; (**F**) pS9-GSK3β; (**G**) pTpS421⁄424-p70S6K; and (**H**) pT246-PRAS40, and relative levels of phosphorylated (**I**) Akt; (**J**) GSK-3β; (**K**) p70S6K; and (**L**) PRAS40 using multiplex bead-based targeted ELISA panels. Immunoreactivity is expressed in fluorescent light units corrected for protein input. Inter-group comparisons were made by Student *t*-tests.

We extended our investigations by examining the effects of chronic ethanol feeding on signaling downstream through the Akt pathway, which regulates cell survival, structure, metabolism, and glucose utilization. We performed multiplex ELISAs to measure Akt, GSK-3β, p70S6K, PRAS40, pS473-Akt, pS9-GSK3β, pTpS421⁄424-p70S6K, and pT246-PRAS40 in gastrocnemius muscle. Chronic ethanol exposed samples had significantly reduced levels of GSK-3β, p70S6K, and pS473-Akt, and calculated ratios of pS9-GSK3β/total GSK-3β and pTpS421⁄424-p70S6K/total p70S6K (Figure 3). In contrast, there were no significant inter-group differences with respect to Akt, PRAS40, pS9-GSK3β, pTpS421⁄424-p70S6K, or pT246-PRAS40 expression. Therefore, chronic ethanol feeding inhibited signaling through Akt and p70S6K, while increasing GSK-3β activity by reducing the relative levels of GSK3β phosphorylation. Note that GSK-3β activity is inhibited by Ser-9 phosphorylation. 

### 3.6. Effects of Chronic Ethanol Exposure on Acetylcholine Homeostasis

**Figure 4:**
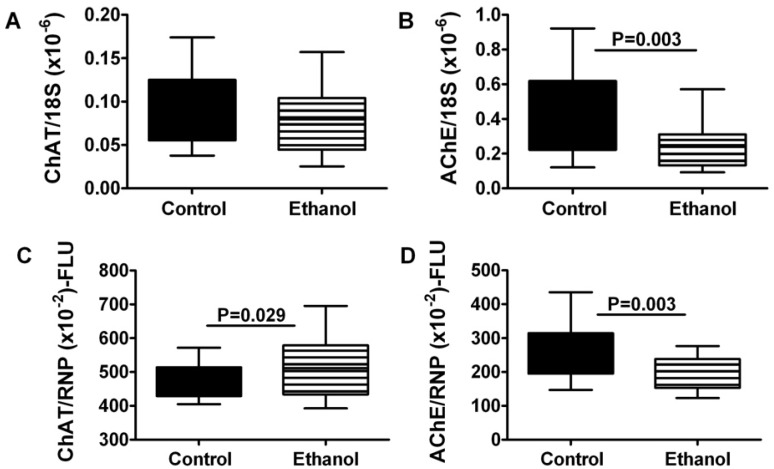
Chronic ethanol feeding impairs expression of acetylcholinesterase in skeletal muscle. Gastrocnemius muscles from control and chronic ethanol fed rats were used to measure (**A**,**B**) mRNA expression or (**C**,**D**) immunoreactivity to (**A**,**C**) choline acetyltransferase (ChAT) and (**B**,**D**) acetylcholinesterase. mRNA was measured by qRT-PCR analysis with results normalized to 18S rRNA, and immunoreactivity was measured using a direct binding duplex ELISA in which large ribonuclear protein expression was used to normalize the levels of ChAT and AChE proteins. Inter-group comparisons were made with Student *t*-tests.

Acetylcholine is a major neurotransmitter used by skeletal muscle. Cholinergic function is regulated by insulin/IGF signaling as well as oxidative stress [36]. To further characterize the effects of chronic ethanol exposure on skeletal muscle function, we measured choline acetyltransferase (ChAT) and acetylcholinesterase (AChE) expression in gastrocnemius muscle by qRT-PCR and direct binding ELISA. Chronic ethanol exposure had no significant effect on ChAT mRNA, although it significantly increased ChAT immunoreactivity (Figure 4). In contrast, chronic ethanol feeding significantly reduced gastrocnemius muscle levels of AChE mRNA and protein.

### 3.7. Consequences of Chronic Ethanol Exposure on Mitochondrial Function and Oxidative Stress in Skeletal Muscle

**Figure 5:**
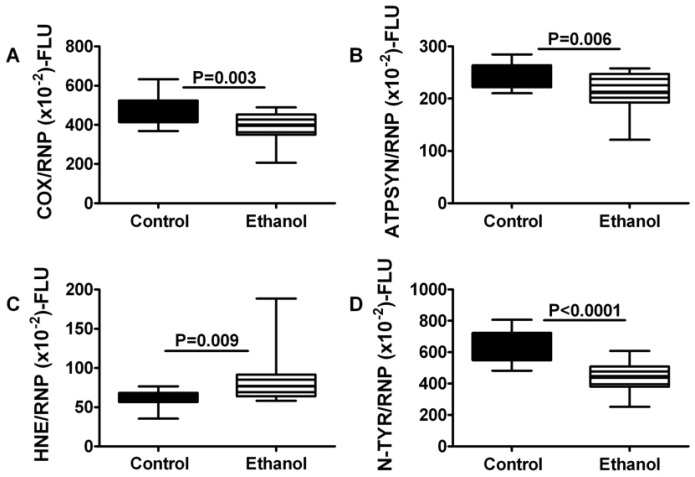
Chronic ethanol feeding impairs mitochondrial oxidative phosphorylation and increases oxidative stress in skeletal muscle. Gastrocnemius muscles from control and chronic ethanol fed rats were used to measure immunoreactivity to (**A**) Complex IV, cytochrome c oxidase (COX); (**B**) ATP synthase (ATPSYN); (**C**) 4-hydroxy-2-nonenal (HNE); and (**D**) 3-nitrotyrosine (NTyR) with a direct binding duplex ELISA in which large ribonuclear protein (RPLPO) expression was used to normalize results. Inter-group comparisons were made with Student *t*-tests.

Important functions regulated by insulin and IGF signaling include energy metabolism and cellular stress. To determine the degree to which these functions are impaired by chronic ethanol exposure, we measured mitochondrial Cytochrome oxidase 4 (COX; Complex IV), ATP synthase (Complex V), 3-nitrotyrosine (*N*-TyR), and 4-hydroxy-2-nonenal (HNE) in gastrocnemius muscle by direct binding ELISA. Chronic ethanol feeding significantly reduced the mean levels of Complexes IV and V, and also nitrotyrosine, but increased the mean level of HNE in skeletal muscle (Figure 5).

## 4. Discussion

### 4.1. General Comments

The principal goals of this study were to determine if alcohol-related myopathy could be produced experimentally by chronic administration of relatively high levels of ethanol, and assess the degree to which the molecular and biochemical features of alcohol-related myopathy correspond to those associated with alcohol-induced liver and brain degeneration, *i.e*., insulin/IGF resistance with impaired Akt pathway activation and increased oxidative stress [37]. Although our chronic ethanol-feeding model has been well characterized with respect to liver and brain degeneration and dysfunction [22,23], the presence, nature, and mechanisms of skeletal myopathies have not been described. Our major finding was that chronic heavy ethanol exposure, in the absence of nutritional deficiencies or other toxin exposures caused myopathic myopathy with features shared by alcoholic liver and brain diseases. The pair-feeding with isocaloric diets ensured similar nutritional intake and weight gain for rats in each group. The results suggest that chronic alcohol-induced myopathy is mediated by broad impairments of insulin/IGF signaling through Akt, as well as increased oxidative stress. 

### 4.2. Roles of Impaired Insulin/IGF/IRS Signaling

Using qRT-PCR, we established that skeletal muscle expresses the full spectrum of insulin and IGF polypeptide and receptor genes, as well as IRS-1 and IRS-2. Although IGF polypeptide genes were found to be most abundant, insulin receptor was more abundantly expressed than IGF-1 and IGF-2 receptors. These observations suggest that locally produced IGF trophic factors and systemically (pancreas) derived insulin, participate in the regulation of skeletal muscle structure and function. Moreover, the findings suggest that both IRS-1 and IRS-2 have roles in transmitting signals downstream from the insulin and/or IGF receptors in skeletal muscle. It is noteworthy that very low levels of insulin polypeptide mRNA were measured in skeletal muscle. Local extra-pancreatic expression of insulin mRNA has been reported for liver and brain [22,23]. We hypothesize that this phenomenon may enable tissues with high metabolic demands to selectively fine-tune metabolic functions on demand.

Chronic ethanol exposure broadly inhibited expression of polypeptide, receptor, and insulin receptor substrate genes, which are needed for insulin and IGF signaling in muscle. Therefore, chronic ethanol exposure could impair a large number of functions in skeletal muscle. Skeletal muscle plays a major role in systemic glucose regulation. Correspondingly, in Type 2 diabetes mellitus, skeletal muscle insulin resistance is a fundamental abnormality such that enhancement of insulin sensitivity in skeletal muscle reduces peripheral insulin resistance. Chronic alcohol abuse leads to insulin and IGF resistance in target organs, including liver, brain, and placenta [22,23,24,30,36,38], and it is also associated with peripheral insulin resistance [39], mimicking effects of Type 2 diabetes mellitus. Therefore, impaired insulin receptor expression and function in skeletal muscle could represent an important factor contributing to ethanol-induced peripheral insulin resistance. Moreover, since IGF-1 signaling has an important role in maintaining skeletal muscle structure [29], reduced IGF-1 polypeptide and receptor gene expression could account for the myofiber atrophy in chronic ethanol-fed rats, and possibly also in humans with chronic alcoholic myopathy. 

Over the past several years, emerging data have highlighted the roles of impaired insulin and IGF signaling and increased oxidative stress in the pathogenesis of alcohol-related diseases of the liver and brain [23,40]. Once established, these pathophysiologic processes contribute to progressive cell loss, degeneration, and impairments in organ/tissue function. In addition, since insulin and IGF stimulate energy metabolism, mitochondrial function, cellular homeostasis, growth, repair, motility, survival, and protein expression, impairments in their corresponding intracellular signaling pathways lead to increased oxidative stress, DNA damage, and lipid peroxidation [23,24]. An established pathophysiological effect of chronic alcohol exposure in a number of different organs and tissues, including liver, adult brain, developing brain, and placenta, is insulin/IGF resistance mediated by varying degrees of decreased ligand-receptor binding, decreased phosphorylation and activation of receptor tyrosine kinases, decreased expression of ligands, receptors, and/or IRS molecules, impaired downstream signaling with inhibition of Akt and activation of GSK-3β, and increased activity of phosphatases that regulate positive downstream signaling. The findings herein are consistent with previous studies demonstrating how ethanol mediates its inhibitory effects on organ, tissue, and cellular functions, and illustrate that the inhibitory effects of ethanol on insulin/IGF signaling pathways in skeletal muscle are quite broad due to significantly reduced expression of trophic factors, receptors, and IRS genes. In addition, the studies herein suggest that increased oxidative stress, which itself promotes insulin/IGF resistance, contributes to the pathogenesis of chronic ethanol induced myopathy.

We performed multiplex ELISAs to further characterize the effects of ethanol on constitutive activation of the insulin and IGF pathways and found that chronic ethanol exposure reduced skeletal muscle expression of insulin receptor and pS312-IRS-1. These results support the qRT-PCR data and provide additional evidence that ethanol impairs insulin signaling at the receptor level. However, the multiplex ELISA studies did not demonstrate ethanol-associated reductions in IGF-1 receptor or IRS-1 immunoreactivity, which is contrary to the qRT-PCR results. These discrepancies might be explained by the greater sensitivity of qRT-PCR compared with ELISAs. Alternatively, post-transcriptional regulatory factors may mediate these effects by stabilizing or reducing turnover of these proteins.

### 4.3. Ethanol Inhibits Akt Pathways in Skeletal Muscle-Potential Role in Mediating Oxidative Stress, Lipid Peroxidation, and Mitochondrial Dysfunction

With regard to pathways downstream of insulin and IGF-1 receptors and IRS, the multiplex ELISA studies demonstrated that chronic ethanol exposure significantly reduced the mean levels of GSK-3β, p70S6K, pS473-Akt, pS9-GSK3β/total GSK-3β, and pTpS421⁄424-p70S6K/total p70S6K in skeletal muscle. Therefore, chronic ethanol feeding impaired insulin/IGF/IRS signaling through Akt and p70S6K, and activated GSK-3β, consistent with previous observations in liver, brain, and placenta [22,23,36,38,41,42]. Inhibition of signaling through Akt, which has important roles in growth, survival, and energy metabolism, could contribute to skeletal muscle atrophy and weakness associated with alcoholic myopathy. In addition, the combined effects of decreased Akt and increased GSK-3β activity would promote oxidative stress and mitochondrial dysfunction. Correspondingly, we detected reduced levels of mitochondrial Complexes IV and V, and increased HNE immunoreactivity in ethanol-exposed skeletal muscle. Again, the findings are reminiscent of those reported with respect to ethanol’s effects on liver and brain [43,44]. The ethanol-associated inhibition of p70S6K is noteworthy because p70S6K mediates micronutrient utilization and protein synthesis. Therefore, inhibition of p70S6K may have contributed to the ethanol-mediated impairments in energy metabolism.

### 4.4. Consequences of Chronic Ethanol Exposure on Cholinergic Function in Skeletal Muscle

Other potential consequences of impaired insulin/IGF signaling include deficits in cholinergic function, as demonstrated previously in neuronal cells [36]. Since acetylcholine is one of the major neurotransmitters utilized by skeletal muscle, it was of interest to examine the effects of ethanol on choline acetyltransferase (ChAT) and acetylcholinesterase (AChE) expression. In contrast to previous findings [36,42,45], we did not detect significant reductions in ChAT mRNA or protein. Instead, we observed significantly reduced levels of AChE mRNA and protein in skeletal muscle of ethanol-fed rats. Therefore, the adverse effects of chronic ethanol exposure on cholinergic function in skeletal muscle are likely mediated by inhibition of AChE rather than ChAT. Since AChE is inhibited by oxidative stress [46,47,48,49], we postulate that ethanol-mediated impairment of insulin/IGF signaling and mitochondrial function, together with increased lipid peroxidation (HNE) promote chronic oxidative stress, which leads to inhibition of AChE expression in skeletal muscle. Previous studies demonstrated that inhibition of AChE is sufficient to cause myofiber atrophy and degeneration [50]. Therefore, alcohol-related myopathy with myofiber atrophy and degeneration are likely mediated by two major factors: (1) broad-ranging impairment of insulin/IGF signaling through Akt pathways, with reduced biosynthetic, repair and growth functions; and (2) increased oxidative stress caused by inhibition of energy metabolism and direct toxic effects of ethanol or its metabolites leading to inhibition of AChE expression.

## 5. Conclusions

In conclusion, this study demonstrates that the Long Evans rat model of chronic ethanol feeding is suitable for investigating mechanisms of alcohol-related myopathy. Moreover, the findings indicate that alcohol-related myopathy is characterized by myofiber atrophy with broad impairments in insulin and IGF signaling mechanisms, including downstream pathways through IRS, Akt, and p70S6K, as well as increased activation of GSK-3β. In addition, alcohol-related myopathy is associated with increased oxidative stress with evidence of mitochondrial dysfunction and attendant inhibition of AChE expression. These results suggest that alcohol-related myopathy is mediated by mechanisms similar to those that cause alcoholic steatohepatitis and neurodegeneration. The consequences of these adverse effects of ethanol on skeletal muscle may extend beyond the obvious in terms of motor weakness, increased proneness to accidents, and disability, since skeletal muscle plays a very important role in regulating peripheral insulin responsiveness, and peripheral insulin resistance is a recognized consequence of chronic ethanol exposure. Given that the mechanisms of alcohol-related myopathy are shared with those that mediate alcohol-related liver and brain degeneration, in future studies it would be worthwhile determining whether specific aspects of skeletal muscle function could be used to gauge long-term adverse systemic effects of chronic ethanol misuse, including degenerative effects in liver and brain.

## Supplementary Files

Supplementary File 1: PDF-Document (PDF, 389 KB)
