# Supplementary material for: Impaired Insulin/IGF Signaling in Experimental Alcohol-Related Myopathy
Source: Nutrients. 2012 Aug 20;4(8):1058–75. doi: 10.3390/nu4081058 (PMC3448087; doi:10.3390/nu4081058)

Supplementary Information

1. Control and Ethanol Containing Liquid Diets Used to Generate the Experimental Models

Adult male (~200–250 g) Long Evans rats (Harlan Sprague Dawley, Inc., Indianapolis, Indiana) were pair-fed with isocaloric liquid diets (BioServ, Frenchtown, NJ) containing 0% (N = 8) or 37% (N = 13) ethanol for 8 weeks. In the F1258 diet, ethanol replaces carbohydrates to balance caloric intake. The F1258 and F1259 diets contained the same concentrations of amino acids, fatty acids, minerals, and vitamins.

Table S1. Rodent liquid diet for pair-feeding to generate chronic alcohol exposure models.

Component	F1259 Control diet (kcal/L)	F1258: Ethanol diet (kcal/L)
Protein	151	151
Fat	359	359
Carbohydrate	490	135
Ethanol	0	355
Total	1000	1000

2. Duplex Enzyme-Linked Immunosorbant Assay (ELISA) Reagents

Duplex ELISAs were performed using commercially available monoclonal or polyclonal antibodies to assess effects of chronic ethanol exposure on skeletal muscle expression of choline acetyltransferase, acetylcholinesterase, mitochondrial enzyme proteins, and indices of oxidative stress. After probing to measure immunoreactivity using Amplex Red horseradish peroxidase-based fluorophore, proteins were re-probed to measure large ribonuclear protein (control protein). Immunoreactivity was detected using an alkaline phosphatase-based fluorophore. Assays were performed in quadruplicate using at least 8 samples per group.

Table S2. Antibodies used in duplex ELISA studies.

Protein target	Abbreviation	Antibody Source	Species	Target
Choline acetyltransferase	ChAT	AbCam	Rabbit polyclonal	Choline acetyltransferase
Acetylcholinesterase	AChE	AbCam	Mouse monoclonal	Acetyl-cholinesterase
Cytochrome C oxidase, Complex IV	COX	Molecular Probes/Invitrogen	Mouse monoclonal	Complex IV
ATP Synthase, Complex V	ATP-Syn	Molecular Probes/Invitrogen	Mouse Monoclonal	Complex V
3-Nitrotyrosine	3-NTyR	Molecular Probes/Invitrogen	Mouse monoclonal	Nitrotyrosine
4-hydroxy-2-nonenal	4-HNE	Percipio Biosciences Inc.	Mouse Monoclonal	Modified 4-HNE
Large ribonuclear protein (P0)	RPLPO	Proteintech Group, Inc.	Rabbit polyclonal	34 kD RPLPO

3. Myofiber Atrophy Caused by Chronic Ethanol Feeding in an Experimental Animal Model

Adult male Long Evans rats were chronically fed with isocaloric liquid diets containing 0% (control) or 37% ethanol by caloric content. After 8 weeks, gastrocnemius muscles were harvested, fixed, and

embedded in paraffin. H&E stained histological sections were used for image analysis to measure myofiber diameter and cross-sectional area (mean ± S.D. [95% confidence interval limits of means]), and determine the percentages of myofibers with central nuclei. At least 200 myofibers per specimen were measured and counted. Inter-group comparisons were made using the Student *T*-test.

Table S3. Experimental Chronic Ethanol Feeding Causes Myopathy.

Variable	Control	Ethanol	*P*-Value
Gastrocnemius fiber diameter (μM)	44.97 ± 5.31 [40.53; 49.41]	25.34 ± 4.38 [19.9; 30.78]	0.0001
Gastrocnemius fiber area (μM^2)	2338.64 ± 643.04 [1728; 2958]	695.08 ± 163.35 [468.3; 921.8]	0.0005
Myofibers with central nuclei (%)	0.81 ± 0.67 [0.25; 1.37]	1.41 ± 0.86 [0.69; 2.12]	0.07

4. Histopathologic Features of Experimental Alcohol-Related Myopathy

Histological studies of gastrocnemius muscles revealed relatively uniform myofiber populations in control samples. Muscles from chronic ethanol fed rats exhibited polygonal myofiber atrophy or hypertrophy, increased central nuclei, and myofiber splitting. There was no inflammation or myofiber necrosis. Paraffin-embedded histological sections of gastrocnemius muscle were stained with H&E and photographed at 200× magnification.

Figure S1. (**A**) Control muscle with regular polygonal myocytes and peripherally placed nuclei. (**B–D**) Ethanol-exposed muscle with marked variability in myofiber size due to atrophy (arrows) and hypertrophy (arrowheads). (**D**) Note increased central nuclei (upper left; circle-also in (**C**), vacuoles (upper right; arrowhead), and myofiber splitting (lower two panels) in the ethanol-exposed muscle. The clear spaces surrounding individual myofibers is artefactual and resulted from Histofix fixation.

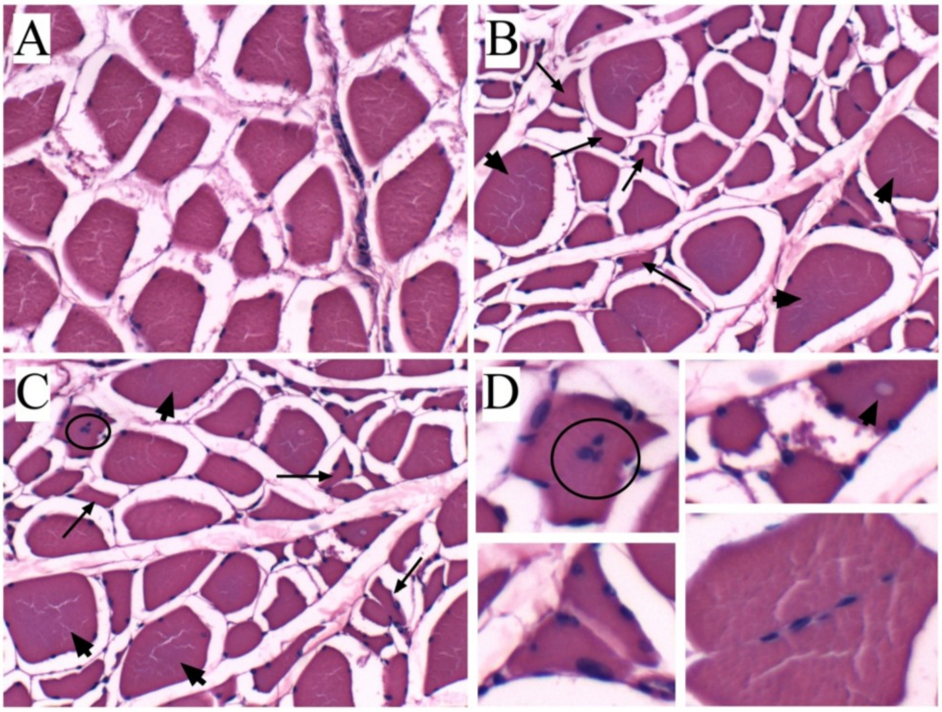

Supplement: Supplementary File 1: — PDF-Document (PDF, 389 KB) [file nutrients-04-01058-s001.pdf]
